# Ten‐year immune persistence and safety of the HPV‐16/18 AS04‐adjuvanted vaccine in females vaccinated at 15–55 years of age

**DOI:** 10.1002/cam4.1155

**Published:** 2017-10-05

**Authors:** Tino F. Schwarz, Andrzej Galaj, Marek Spaczynski, Jacek Wysocki, Andreas M. Kaufmann, Sylviane Poncelet, Pemmaraju V. Suryakiran, Nicolas Folschweiller, Florence Thomas, Lan Lin, Frank Struyf

**Affiliations:** ^1^ Central Laboratory and Vaccination Centre Klinikum Würzburg Mitte Standort Juliusspital Würzburg Germany; ^2^ NZOZ Vitamed Bydgoszcz Poland; ^3^ Faculty of Medicine and Health Sciences University of Zielona Gora Zielona Gora Poland; ^4^ Department of Preventive Medicine Poznan University of Medical Sciences Poznan Poland; ^5^ Department of Gynecology Charité‐Universitätsmedizin Berlin Berlin Germany; ^6^ GSK Rixensart Belgium; ^7^ GSK Bangalore India; ^8^ GSK Wavre Belgium

**Keywords:** AS04‐HPV‐16/18 vaccine, older women, persistence, safety

## Abstract

Women remain at risk of human papillomavirus (HPV) infection for most of their lives. The duration of protection against HPV‐16/18 from prophylactic vaccination remains unknown. We investigated the 10‐year immune response and long‐term safety profile of the HPV‐16/18 AS04‐adjuvanted vaccine (AS04‐HPV‐16/18 vaccine) in females aged between 15 and 55 years at first vaccination. Females who received primary vaccination with three doses of AS04‐HPV‐16/18 vaccine in the primary phase‐III study (NCT00196937) were invited to attend annual evaluations for long‐term immunogenicity and safety. Anti‐HPV‐16/18 antibodies in serum and cervico‐vaginal secretions (CVS) were measured using enzyme‐linked immunosorbent assay (ELISA). Serious adverse events (SAEs) were recorded throughout the follow‐up period. Seropositivity rates for anti‐HPV‐16 remained high (≥96.3%) in all age groups 10 years after first vaccination. It was found that 99.2% of 15–25‐year olds remained seropositive for anti‐HPV‐18 compared to 93.7% and 83.8% of 26–45‐year olds and 45–55‐year olds, respectively. Geometric mean titers (GMT) remained above natural infection levels in all age groups. Anti‐HPV‐16 and anti‐HPV‐18 titers were at least 5.3‐fold and 3.1‐fold higher than titers observed after natural infection, respectively, and were predicted to persist above natural infection levels for ≥30 years in all age groups. At Year 10, anti‐HPV‐16/18 antibody titers in subjects aged 15–25 years remained above plateau levels observed in previous studies. Correlation coefficients for antibody titers in serum and CVS were 0.64 (anti‐HPV‐16) and 0.38 (anti‐HPV‐18). This study concluded that vaccinated females aged 15–55 years elicited sustained immunogenicity with an acceptable safety profile up to 10 years after primary vaccination, suggesting long‐term protection against HPV.

## Introduction

Persistent infection with an oncogenic human papillomavirus (HPV) type is a prerequisite for developing cervical cancer (CC) 1. The latter is the fourth most common cancer in females worldwide 2. To date, at least 13 HPV types have been defined as oncogenic. Among them, HPV‐16 and HPV‐18 are estimated to contribute to 71% of invasive CC cases 3.

The AS04‐HPV‐16/18 vaccine (*Cervarix*, GSK, Belgium) is licensed for administration from the age of 9 years in most countries, with an upper age limit varying from 25 years to no limit 4, 5. Current vaccination programs worldwide target young adolescents before they become sexually active and exposed to HPV. Nevertheless, due to the continuous risk of acquiring new infections throughout a woman's active sexual life, CC screening and vaccination against HPV in older women could also be effective against development of CC 6, 7, 8. The primary phase‐III study (NCT00196937) demonstrated that the AS04‐HPV‐16/18 vaccine was immunogenic and well tolerated in females aged 15–55 years 9. The follow‐up study showed that anti‐HPV‐16/18 antibodies in serum and cervico‐vaginal secretions (CVS) persisted for 6 years after vaccination in this population with an acceptable safety profile 10. In a study involving women above 25 years of age, the vaccine also demonstrated protection against HPV‐16 and HPV‐18 persistent infections, cytological abnormalities, and CIN1+  (NCT00294047) 11.

As the exact duration of protection against HPV‐16 and ‐18 following the HPV‐16/18 AS04‐adjuvanted vaccine administration was unknown at licensure, long‐term follow‐up studies were conducted. This study evaluated the 10‐year immune persistence and long‐term safety of the vaccine in women aged 15–55 years at the time of the first vaccination.

## Material and Methods

### Study design and subjects

The primary, open, phase‐III, age‐stratified study, which was conducted at three centers in Germany and Poland, vaccinated females aged 15–55 years with three doses of the AS04‐HPV‐16/18 vaccine according to a 0‐, 1‐, and 6‐month schedule (NCT00196937). All subjects who received three doses of the vaccine in the primary study were invited to return for annual immunogenicity and safety evaluations from Year 5 onward. This study was designed to provide 10‐year follow‐up of immunogenicity and safety (NCT00947115) in these subjects. Females who missed an annual visit remained eligible for inclusion at a later time point.

Subjects were not enrolled in the study if they had received previous vaccination against HPV other than the AS04‐HPV‐16/18 vaccine, or chronic administration of immunosuppressants, immunoglobulins, or blood products less than 3 months prior to blood sampling.

The study was conducted in accordance with the Declaration of Helsinki, principles of Good Clinical Practice (GCP), and all applicable regulatory requirements. Written informed consent was obtained from each subject before conducting any study‐specific procedures.

### Immunogenicity assessment

At each annual visit (Years 5, 6, 7, 8, 9, and 10), a 10‐mL sample of whole blood was taken from each subject and CVS samples were also collected in consenting subjects who volunteered for this additional procedure as already described in the primary publication 9. The presence of anti‐HPV‐16/18 antibodies were evaluated using an enzyme‐linked immunosorbent assay (ELISA). Overtime stability of the validated HPV‐16/18 ELISA assays on serum and CVS was monitored by in process controls tested on each ELISA plate and a quality control panel testing every 6 months. This monitoring enables the direct comparison of antibody levels across studies. Until Year 7, seropositivity was defined as antibody titers ≥8 EU/mL and ≥7 EU/mL for anti‐HPV‐16 and anti‐HPV‐18 antibodies, respectively. While monitoring the quality of the assay, a high variability was observed in the low range of assay results from unvaccinated trial participants. From Year 8 onward, the precision of the assay was therefore increased by changing the seropositivity cut‐offs to antibody titers ≥19 EU/mL and ≥18 EU/mL for anti‐HPV‐16 and anti‐HPV‐18, respectively. Subjects with antibody titers below the assay cut‐off were assigned an arbitrary GMT value of half the cut‐off for the purpose of GMT calculations.

### Safety assessment

Long‐term safety of the AS04‐HPV‐16/18 vaccine up to 10 years after first vaccination was evaluated. The analysis of safety was based on the TVC comprising all vaccinated subjects who received three doses of the AS04‐HPV‐16/18 vaccine in the primary study and participated in the Year‐10 extension study.

A serious adverse event (SAE) was defined as any untoward medical event that resulted in death, was life‐threatening, required hospitalization or prolongation of existing hospitalization, or resulted in disability/incapacity. Medical or scientific judgment was exercised in deciding whether reporting was appropriate in other situations such as important medical events that may not have been immediately life‐threatening or resulted in death or hospitalization but may have jeopardized the subject or may have required medical or surgical intervention. In such cases, these events were also considered serious. Any vaccine‐related, study participation‐related, or GSK concomitant medication‐related SAEs or fatal SAEs were recorded throughout the study period.

### Statistical analyses

The primary endpoint was the evaluation of long‐term immunogenicity of the HPV‐16/18 vaccine in serum from all vaccinated subjects by ELISA at each time point (Years 5, 6, 7, 8, 9, and 10) following the first vaccine dose. The according‐to‐protocol (ATP) cohort for analysis of immunogenicity included all evaluable subjects, that is, subjects included in the ATP immunogenicity analysis during the primary study (NCT00196937), who met all eligibility criteria, complied with the study procedures defined in the protocol, had no elimination criteria during the study and in whom serology results were available.

Seropositivity rates and geometric mean titers (GMTs) were calculated with exact 95% confidence intervals (CIs) for anti‐HPV‐16/18 antibodies. Secondary analyses compared the immune response of subjects to the AS04‐HPV‐16/18 vaccine in this study (NCT00947115) with the immunogenicity plateau levels measured in the efficacy study (NCT00689741) and associated long‐term follow‐up studies (NCT00120848, NCT00518336) 12, 13, 14, and the immune response measured after natural infection in subjects from the PATRICIA study (NCT00122681) 15, in which efficacy was demonstrated in women aged 15–25 years 16. GMT values from CVS samples up to 10 years after first vaccination were evaluated. CVS samples with Hemastix values ≥200 erythrocytes per *μ*L were excluded from antibody testing as they were considered to be contaminated with blood. The analysis of immunogenicity of CVS samples was based on the total vaccinated cohort (TVC) that was comprised of subjects who volunteered for CVS sampling and for whom CVS results were available. Anti‐HPV‐16/18 antibody titers were determined in both serum and CVS samples. Since antibody levels fluctuate during menstrual cycles, individual anti‐HPV‐16 and anti‐HPV‐18 titers were divided by the total IgG concentration, to evaluate the correlation between vaccine‐induced IgG titers in serum and CVS 9, 17, 18.

### Mathematical modeling

Post hoc mathematical modeling was conducted to evaluate long‐term immunogenicity against HPV‐16 and HPV‐18 for at least 30 years after vaccination. Two different mixed effects models were used, piecewise and modified power‐law (Fraser) 19. The piecewise model used a linear function in which the data were fitted on four non‐overlapping time intervals (with breakpoints at months 7, 12, 21, and 48) that corresponded to the observed decay of humoral antibodies. The previous modeling with 6‐year data included three breakpoints (months 7, 12, and 21) in the piecewise model that predicted population antibody responses above the natural infection antibody level for up to 20 years 20. As our results provided data for up to 10 years, one more piece or breakpoint was defined for the piecewise model. The selection of the additional breakpoint, that is, month 48, was based on the Akaike Information Criteria 21. The modified power‐law method that estimated antibody decay over time after vaccination was based on the dynamic populations of B‐cells (activated and memory B‐cells) that account for long‐term persistence and antibody plateau. The piecewise and modified power‐law models were fitted using MIXED and NLMIXED statistical analyses system procedures, respectively.

## Results

### Subject disposition and demographics

Of 666 subjects from Germany and Poland who received three doses of the AS04‐HPV‐16/18 vaccine in the primary study, 524 participated in the trial and were included in the TVC cohort for safety analysis. Among them, 470 completed the Year 10 assessment, that is, Year 10 TVC, and 451 were included in the Year 10 ATP cohort for immunogenicity (Fig. S1). Subjects were stratified according to age and the TVC cohort included 142 subjects in the 15–25‐year age group, 172 in the 26–45‐year age group, and 156 in the 46–55‐year age group. Mean age at Year 10 was 30.6 years (standard deviation, SD: 2.8), 46.1 years (SD: 6.0) and 59.6 years (SD: 2.8) in the three age groups, respectively. The population was predominantly Caucasian.

### Immunogenicity

Seropositivity rates for anti‐HPV‐16 remained high (≥96.3%) in all age groups 10 years after first vaccination (Table 1). For anti‐HPV‐18, 99.2% of subjects in the 15–25‐year age group remained seropositive compared with 93.7 and 83.8% in the 26–45 and 46–55‐year age groups, respectively.

**Table 1:** Percentage of seropositive subjects for anti‐HPV‐16 and anti‐HPV‐18 antibodies in each age group at Year 10 in initially seronegative subjects

Antibody	Age groups (years)								
	[15–25]			[26–45]			[46–55]		
	%	95% CI		%	95% CI		%	95% CI	
		LL	UL		LL	UL		LL	UL
Anti‐HPV‐16	100	97.0	100	99.2	95.5	100	96.3	90.7	99.0
Anti‐HPV‐18	99.2	95.7	100	93.7	88.3	97.1	83.8	76.4	89.7

95% CI: 95% confidence interval.

LL, Lower limit; UL, Upper limit; HPV, Human Papillomavirus.

**Figure 1:**
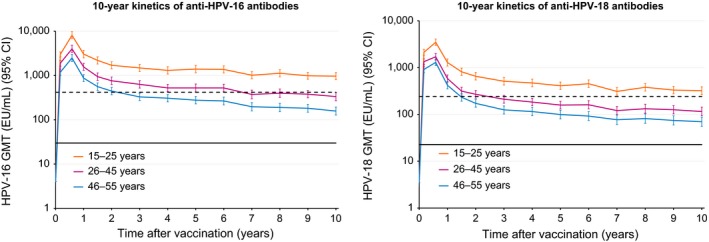
Long‐term kinetics of anti‐HPV‐16 (left) and anti‐HPV‐18 (right) antibodies (According to protocol [ATP] cohort for immunogenicity). Plateau levels (dashed lines) are 418.3 EU/mL and 242.6 EU/mL for HPV‐16 and HPV‐18, respectively (month 107–113 in reference study) 13, 14, 16. Natural infection levels (solid lines) are 29.8 EU/mL and 22.7 EU/mL for HPV‐16 and HPV‐18, respectively 15. CI, confidence interval; GMT, geometric mean titer; HPV, human papillomavirus.

Serum anti‐HPV‐16 antibody levels at Year 10 appeared to be lower [non‐overlapping CIs] in the older group (157.4 EU/mL, 95% CI: 128.4; 193.1) compared with the 26–45 and 15–25‐year age groups, that is, 334.4 EU/mL (95% CI: 270.5; 413.5) and 965.4 EU/mL (802.2; 1161.8), respectively [non‐overlapping CIs]. A similar pattern of change was seen with serum HPV‐18 antibody levels as shown in Table 2, that is, 69.7 EU/mL (95% CI: 56.0; 86.8) in the 46–55‐year age group, 115.4 EU/mL (95% CI: 93.9; 142.0) in the 26–45‐year age group, and 321.1 EU/mL (95% CI: 265.0; 389.1) in the 15–25 year‐group (non‐overlapping CIs). The antibody kinetics of both anti‐HPV‐16 and anti‐HPV‐18 at Year 10 are shown in Figure 1. Following a peak in antibody levels 1 month after third vaccination, there was a gradual decline for both antigens, which plateaued after 2 years. GMTs in all age groups remained above natural infection levels. Anti‐HPV‐16 and anti‐HPV‐18 antibody titers were at least 5.3‐fold (95% CI: 4.2–6.6) and 3.1‐fold (95% CI: 2.5–3.7) higher than the titers observed after natural infection, respectively. (Table 3)

**Table 2:** Anti‐HPV‐16 and anti‐HPV‐18 serum and cervico‐vaginal secretions antibody levels at Year 10 (serum: According to protocol cohort for immunogenicity, seronegative subjects at baseline; CVS: Total vaccinated cohort for immunogenicity)

Age groups (years)	Serum				CVS^a^			
	GMT	95% CI		*n* (*N*)	GMT	95% CI		*n* (*N*)
		LL	UL			LL	UL	
HPV–16								
[15–25]	965.4	802.2	1161.8	123 (123)	43.4	28.1	67.1	29 (40)
[26–45]	334.4	270.5	413.5	120 (121)	34.3	24.1	48.7	22 (37)
[46–55]	157.4	128.4	193.1	103 (107)	56.0	31.5	99.7	13 (24)
HPV–18								
[15–25]	321.1	265.0	389.1	126 (127)	29.4	17.6	49.4	16 (40)
[26–45]	115.4	93.9	142.0	133 (142)	22.7	13.8	37.3	16 (37)
[46‐55]	69.7	56.0	86.8	109 (130)	45.1	22.9	88.6	8 (24)

a CVS: cervico‐vaginal secretion sample with hemastix < 200 erythrocytes per *μ*L. GMT: Geometric mean titer (EU/mL); 95% CI: 95% confidence interval; LL: Lower limit; UL: Upper limit; HPV: Human Papillomavirus; N: number of subjects with available results; n: number of subject with titre equal to or above the lower limit of quantitation in CVS at 0.58 EU/mL for HPV‐16 and 0.35 EU/mL for HPV‐18 and in serum 19 EU/mL for HPV‐16 and 18 EU/mL for HPV‐18

**Table 3:** Comparison of the elicited immune responses against HPV‐16 and HPV‐18 with the observed geometric mean titers after natural infection, for the three age groups

Age group (years)	GMT^a^	GMT natural infection level^b^	Fold factor		
			Value	95% CI	
				LL	UL
HPV–16					
[15–25]	965.4	29.8	32.4	26.3	39.9
[26–45]	334.4	29.8	11.2	9.1	13.8
[46–55]	157.4	29.8	5.3	4.2	6.6
HPV–18					
[15–25]	321.1	22.7	14.1	11.8	17.0
[26–45]	115.4	22.7	5.1	4.3	6.1
[46–55]	69.7	22.7	3.1	2.5	3.7

a GMTs: Geometric mean titers (EU/mL) for anti‐HPV‐16 antibodies and anti‐HPV‐18 antibodies at Year 10 time point for subjects seronegative before vaccination.

b Geometric mean titer value associated with natural infection^[9]^ . 95% CI: 95% confidence interval; LL: Lower limit; UL: Upper limit; HPV: Human Papillomavirus.

In subjects vaccinated at the age of 15–25, anti‐HPV‐16 antibody titers at Year 10 remained above the plateau levels observed in previous efficacy studies (NCT00689741, NCT00120848, NCT00518336).

At Year 10, anti‐HPV‐16 antibody titers in initially seronegative subjects from the Y10 ATP cohort for immunogenicity were 2.31‐fold (95% CI: 1.76, 3.03) higher in the 15–25‐year age group, 0.80‐fold lower (95% CI: 0.59, 1.07) in the 26–45‐year age group and 0.38‐fold lower (95% CI: 0.28, 0.50) in the 46–55‐year age group compared to those measured at the end of the 113‐month (≈ 9.4‐year) follow‐up in subjects vaccinated at the age of 15–25 years in the efficacy and long‐term follow‐up studies. Anti‐HPV‐18 antibody titers were found to be 1.32‐fold higher (95% CI: 1.00, 1.75) in the 15–25‐year age group, 0.48‐fold lower (95% CI: 0.35, 0.64) in the 26–45‐year age group, and 0.29‐fold lower (95% CI: 0.21, 0.39) in the 46–55‐year age group as compared to the same reference. In older age groups, that is, 26–45 and 46–55, antibody titers were similar to or below the plateau levels for HPV‐16 and HPV‐18 antigens (Fig. 1).

At Year 10, 107 women (15–25 years: 41; 26–45 years: 40; 46–55 years: 26) provided CVS samples with <200 erythrocytes/*μ*L, which were suitable for HPV antibody testing. At this time point, anti‐HPV‐16 antibodies were detected in 70.7 (*n* = 29), 57.5 (*n* = 23), and 53.8% (*n* = 14) of CVS samples of women from the 15–25, 26–45, and 46–55‐year age groups, respectively. Anti‐HPV‐18 antibodies were detected in 39.0, 45.0, and 34.6% of the CVS samples, respectively.

Overall, correlation coefficients for anti‐HPV‐16 and anti‐HPV‐18 antibody titers between serum and CVS samples were 0.64 and 0.38, respectively.

### Mathematical modeling

**Figure 2:**
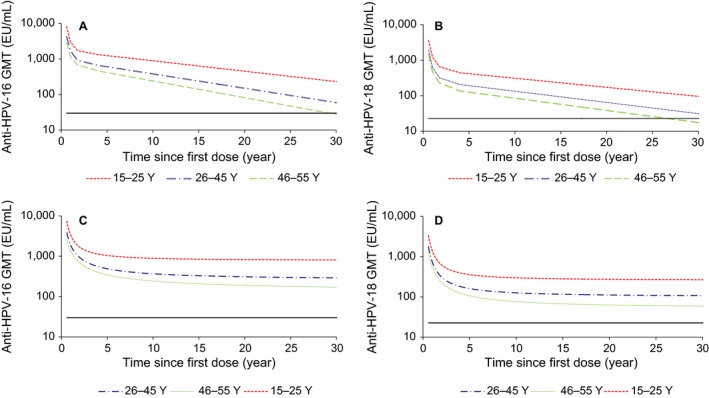
Anti‐HPV‐16/18 antibody titers predicted by the piecewise (A, B) or modified power‐law (C, D) models up to 30 years after the first vaccine dose in subjects who received all three doses of HPV vaccine, by age group. The horizontal line indicates the mean antibody titer associated with natural infection in a previously published study 22. GMT, geometric mean titer.

The results of extrapolating anti‐HPV‐16 and anti‐HPV‐18 antibody titers to at least 30 years after the first vaccine dose are shown in Figure 2. The modified power‐law model predicted that mean antibody titers would remain above the levels associated with natural infection in a previous study in all three age groups 22. The piecewise model, which takes into account a decrease in antibody levels over time, predicted that mean antibody levels would remain above natural infection levels in the two younger age groups, but not in the older, at Year 30. More specifically, this model projected that antibody levels would remain above natural infection levels for 38.8, 17.8, and 12.4 years with anti‐HPV‐16 and 28.8, 11.3, and 4.4 years with anti‐HPV‐18 for 95% of women aged 15–25, 26–45, and 46–55 years, respectively.

The modified power‐law model predicted that anti‐HPV‐16 levels would remain above the natural infection level lifelong in females aged 15–45 years and for up to 31.4 years in those aged 46–55 years. Similarly, this model projected that anti‐HPV‐18 levels would remain above the natural infection level lifelong in females aged 15–25, while the predicted time that 95% of women would still have anti‐HPV‐18 levels above natural infection levels was 25.9 and 3.58 years in the 26–45 and 46–55‐year age groups, respectively (Table 4).

**Table 4:** Predicted anti‐HPV‐16 and anti‐HPV‐18 antibody responses by piecewise and modified power‐law models for the three age groups

Age group (years)	HPV‐16				HPV‐18			
	Piecewise model		Modified power‐law model		Piecewise model		Modified power‐law model	
	Predicted GMT at Year 30 after first dose (EU/mL)	Predicted time (years)^a^	Predicted GMT at Year 30 after first dose (EU/mL)	Predicted time (years)^a^	Predicted GMT at Year 30 after first dose (EU/mL)	Predicted time (years)^a^	Predicted GMT at Year 30 after first dose (EU/mL)	Predicted time (years)^a^
[15–25]	230.759	38.83	863.619	>30	95.849	28.83	282.830	>30
[26–45]	58.875	17.83	340.753	>30	31.232	11.25	120.952	25.92
[46–55]	27.424	12.42	203.413	31.41	<18 (17.47)	4.42	68.239	3.58

GMT, geometric mean titer; HPV, Human Papillomavirus.

a Predicted time ensuring 95% of women will still have predicted titers above natural infection level (29.8 EU/mL for HPV‐16, 22.7 EU/mL for HPV‐18).

### Safety

Two deaths were recorded over the 10‐year follow‐up period due to chronic lymphocytic leukemia and malignant lung neoplasm. None of these fatal SAEs were considered to be related to vaccination by the investigator. One case of cervical dysplasia of moderate severity, which was detected 8.4 years after the first vaccine dose in a 36‐year‐old female, was classified as vaccine‐related SAE by the investigator. Polymerase chain reaction (PCR) on biopsy indicated the presence of high‐risk HPV although the type causing the lesion could not be identified (PCR mix). The subject was HPV‐16 seropositive and of unknown DNA status at baseline. This event had resolved by the end of the study. (Table S1).

## Discussion

### Findings

This is the first study to examine the 10‐year immune persistence and safety of the AS04‐HPV‐16/18 vaccine administered to women up to 55 years of age.

Ten years after receiving the first vaccination, most subjects were still seropositive for HPV‐16/18 in all age groups. Antibody kinetics had similar profiles across all age groups, displaying a decline in GMT values with increasing ages at first vaccination. As previously observed in the same study, a more or less potent correlation was observed between the anti‐HPV‐16/18 antibody levels in the serum and CVS samples, further supporting the previous observation of long‐lasting transudation of serum antibodies across the cervical epithelium, although this association was not as strong as that seen at the Year 6 time point and was quite weak for HPV‐18, presumably as a result of a smaller number of evaluable samples at the Year 10 time point 10, 18, 23.

The long‐term safety profile of the vaccine was clinically acceptable. The occurrence of cervical dysplasia was of unclear clinical significance as the subject had already been infected by HPV‐16 before vaccination and no baseline DNA testing was performed at study entry. It is not known if the lesion was the result of a reactivation or a new infection by a high‐risk HPV type.

### Strengths and limitations

This study confirmed, for the first time, the long‐term persistence of anti‐HPV‐16/18 antibodies in women vaccinated up to the age of 55. The study was further strengthened by the collection of serum and CVS samples that allowed for correlation of antibody titers, although few CVS samples were collected and evaluable at study end. It was limited by the lack of cervical screening at enrolment and the absence of clinical or virological endpoints.

### Interpretation

Ten years after first vaccination, the AS04‐HPV‐16/18 vaccine showed sustained immunogenicity in all age groups. Antibody titers at Year 10 were still at least three times higher than those observed after natural infection across all age groups, and in the older age categories (≥25 years), titers were similar to or just below the plateau levels associated with long‐term efficacy 14, 22. These findings were in line with the results of the primary study and consistent with our expectations, since GMTs are known to decline with increasing age at vaccination 9. These results, along with the sustained efficacy seen in another study for up to 7 years post vaccination in women aged ≥25 years, suggest that the vaccine may confer protection for up to 10 years and possibly longer, even in older subjects 11.

Mathematical modeling predicted detectable anti‐HPV‐16 and anti‐HPV‐18 antibody responses for at least 30 years after the first vaccination. Since women are potentially at risk of exposure to HPV for a long period of time throughout their lives, these findings suggest that the duration of protection against CC conferred by HPV vaccination may be close to lifelong.

## Conclusion

This study confirmed the sustained immunogenicity of the AS04‐HPV‐16/18 vaccine in women aged 15–55 years, with an acceptable safety profile for at least 10 years after primary vaccination. Mathematical modeling predicted that anti‐HPV‐16/18 antibody titers, 30 years post vaccination would still be above natural infection levels in all age groups using a modified power‐law model, and in the 15–25 years and 26–45‐year age groups using a piecewise model. Considering the time required to develop cervical cancer, this suggests that protection may be close to lifelong. Women in age groups not targeted by pediatric or adolescent immunization and catch‐up programs may still individually benefit from HPV vaccination.

## Trademark statement

*Cervarix* is a trademark owned by the GSK group of companies.

## Conflict of Interest

GlaxoSmithKline Biologicals SA took in charge all costs associated with developing and publishing this manuscript. TF Schwarz received personal fees from the GSK group of companies outside the submitted work. L Lin was previously insourced to the GSK group of companies as a consultant and is now an employee of the GSK group of companies. S Poncelet, PV Suryakiran, N Folschweiller, and F Struyf are employees of the GSK group of companies. S Poncelet, L Lin, and F Struyf hold shares in the GSK group of companies as part of their employee remuneration.

## Supporting information


**Table S1**. List of related or fatal SAEs during the entire study (Year 0 to Year 10: Year 10 total vaccinated cohort)

**Figure S1.** Study design flowchart showing subject disposition.
