# Supplementary material for: Ten‐year immune persistence and safety of the HPV‐16/18 AS04‐adjuvanted vaccine in females vaccinated at 15–55 years of age
Source: Cancer Med. 2017 Oct 5;6(11):2723–31. doi: 10.1002/cam4.1155 (PMC5673947; doi:10.1002/cam4.1155)
Supplement: Supplementary file 1 — Table S1. List of related or fatal SAEs during the entire study (Year 0 to Year 10: Year 10 total vaccinated cohort) [file CAM4-6-2723-s001.docx]

**Supplementary Table 1: List of related or fatal SAEs during the entire study (Year 0 to Year 10: Year 10 total vaccinated cohort)**

| **SAE** | **Age group** | **Age at Onset** | **Dose** | **Day of onset** | **Causality** | **Outcome** |
| --- | --- | --- | --- | --- | --- | --- |
| Cervical dysplasia | [26-45] | 36 | 3 | 3,064 | HPV-Related | Resolved |
| Chronic lymphocytic leukemia | [26-45] | 45 | 3 | 1,314 | Not related | Fatal |
| Lung carcinoma | [46-55] | 62 | 3 | 3,077 | Not related | Fatal |

HPV: Human Papillomavirus

SAEs: Serious adverse events
