# Supplementary figures and images for: Ten‐year immune persistence and safety of the HPV‐16/18 AS04‐adjuvanted vaccine in females vaccinated at 15–55 years of age
Source: Cancer Med. 2017 Oct 5;6(11):2723–31. doi: 10.1002/cam4.1155 (PMC5673947; doi:10.1002/cam4.1155)

**Supplementary Figure 1:** Study design flowchart showing subject disposition.

**
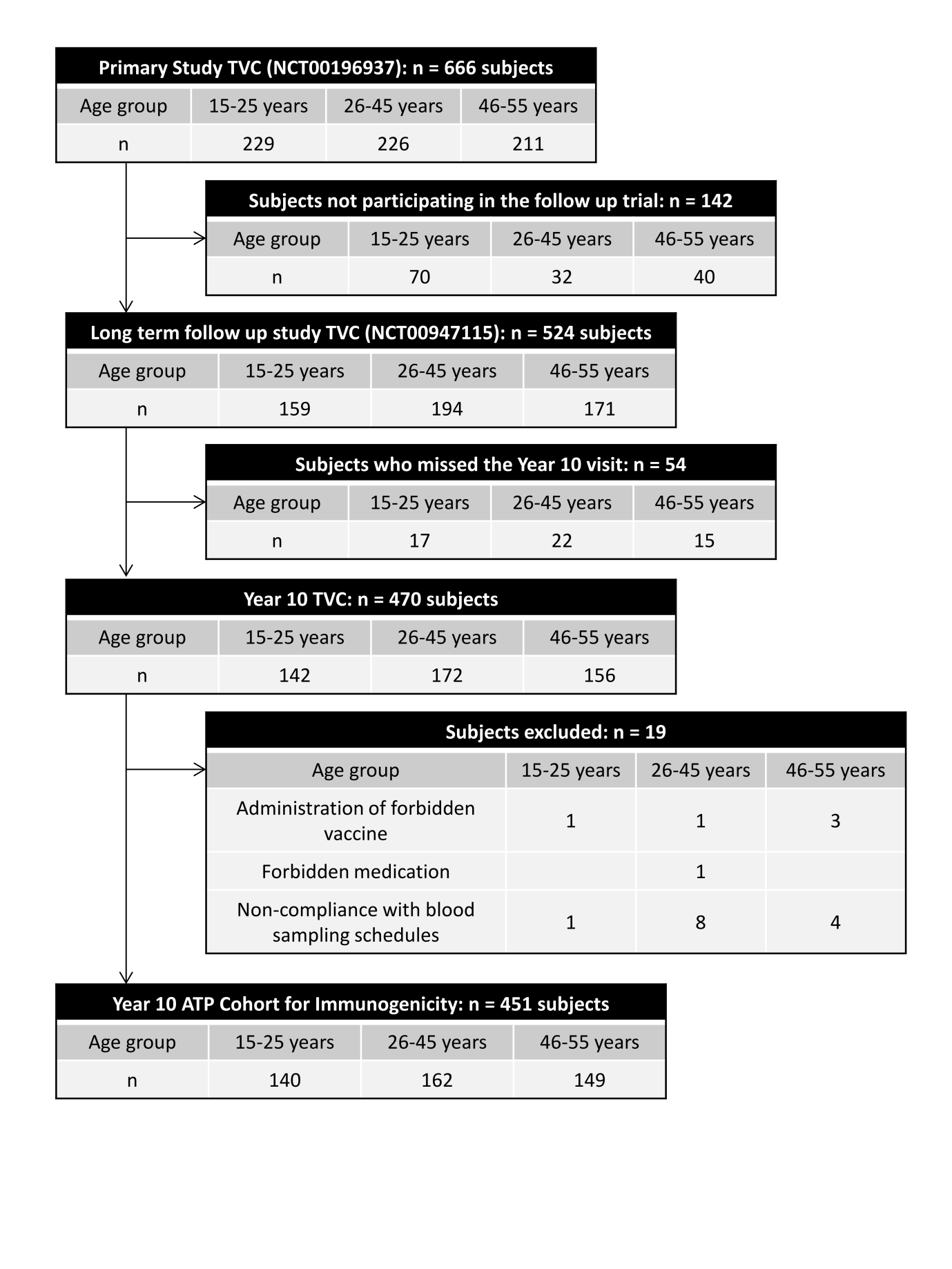
**

Supplement: Supplementary file 2 — Figure S1. Study design flowchart showing subject disposition. [file CAM4-6-2723-s002.docx]
